# Anxiety, Automatic Negative Thoughts, and Unconditional Self-Acceptance in Rheumatoid Arthritis: A Preliminary Study

**DOI:** 10.1155/2014/317259

**Published:** 2014-03-20

**Authors:** Ramona Paloș, Loredana Vîșcu

**Affiliations:** ^1^Department of Psychology, West University of Timişoara, V. Pârvan Street, No. 4, 300323 Timi*ș*oara, Romania; ^2^Social Work Department, University “Eftimie Murgu” of Re*ș*i*ț*a, Train Vuia Square, No. 1-4, 320085 Re*ș*i*ț*a, Romania

## Abstract

*Objectives.* This research was carried out in two stages: the objectives of the first stage were (1) to identify the existing relationships between the level of anxiety, the frequency of automatic negative thoughts, and unconditional self-acceptance and (2) to capture the existing differences regarding these variables between people diagnosed with rheumatoid arthritis and those with no such medical history. * Methods.* The sample made up of 50 subjects filled out the following three questionnaires: the Hamilton Anxiety Scale, the Automatic Thoughts Questionnaire, and the Unconditional Self-Acceptance Questionnaire. * Results.* Psychological anxiety is positively correlated with automatic negative thoughts, while unconditional self-acceptance is negatively correlated with both psychological anxiety and somatic anxiety as well as with automatic negative thoughts. All studied variables were significantly different in rheumatoid arthritis as compared to the control population. * Conclusions.* The results showed the presence to a greater extent of anxiety and automatic negative thoughts, along with reduced unconditional self-acceptance among people with rheumatoid arthritis. Intervention on these variables through support and counseling can lead to reducing anxiety and depression, to altering the coping styles, and, implicitly, to improving the patients' quality of life.

## 1. Introduction

Rheumatoid arthritis (RA) is one of the main and most common inflammatory rheumatic diseases that affect the joints, causing chronic pain and disability [1]. Although in arthritis disability has been associated with damaging effect on the joints, gradually, the predictive nature of psychological and social factors has also been acknowledged in increasing disability over time (e.g., attitude towards the disease, coping styles, anxiety, automatic negative thoughts, or depression). This can determine significant influences in carrying out one's roles, in one's quality of life and mental health [2]. Fortunately, as the disease progresses, patients can learn how to adapt to it and its consequences, so as to maintain a normal level of distress [3].

The literature explaining rheumatoid arthritis highlights the role played by psychological factors in health-seeking behaviors and in using medical services [4] or in developing counseling and support programs.* The self-regulation model *suggests the fact that the emotional and cognitive aspects of perceiving the disease guide the response to this illness and determine the efficiency of the coping mechanisms developed by patients. Coping—seen as a general concept employed to describe cognitive, emotional and behavioral reactions to stressful events and situations—and perception of the disease appear as the most significant variables in the relationship between physical and psychological factors in RA [5]. This is explained by the fact that in the process of coping one can trace three dimensions: (1) one related to the set of cognitions concerning the significance that a person attributes to a stressful event; (2) a second dimension related to the set of cognitions that a person has with regard to resources for coping with a stressful event; (3) and a third dimension referring to avoidance-oriented coping [5, page 204]. In the case of people diagnosed with RA, research studies focused particularly on finding answers to two questions: how these people use coping mechanisms in this disease and why, when faced with the same stressful events, people cope differently. With these patients, psychotherapeutic approaches focused on behavioral therapy, on cognitive therapy, on education for arthritis, on stress management, or on supportive counseling [6].

The* biopsychosocial approach to arthritis* also acknowledged the impact of psychological and social factors on the evolution of arthritis [7]. Among the most important factors that influence coping mechanisms are stress, depression, self-efficacy, lack of hope, and social relationships. A large number of research studies suggest the existence of a link between psychological factors and those that influence pain and disability [8, 9]. Stress and depression can be both considered as consequences of arthritis, but they can also exacerbate arthritis activity [8–10]. Also, anxiety and depression influence pain in different ways and call for different means of intervention [11]. Anxiety, for example, is linked to the perseverative cognition, a maladaptive process of maintaining the cognitive representation of a stressor in the absence of implementing an adaptive behavior of coping [12]. Moreover, automatic negative thoughts, simultaneously occurring with a stressful situation, lead to depression [13, 14]. Although automatic thoughts are an element of both normal and abnormal cognitions, the presence of a consistent pattern of automatic negative thoughts leads to one's reduced ability to function and adequately adapt to the environment [15]. The origin of these thoughts lies in the pessimistic beliefs about oneself, about one's future and relationships with others, and they consist of negative statements about oneself. Certain studies correlate automatic negative thoughts with depression, as well as with one's coping styles, with the problem solving process [16]. Also, another factor that determines personal adaptation and well-being is unconditional self-acceptance [17], seen as an adaptive acceptance of oneself regardless of the approval, respect, or love received from others [18]. Research studies carried out by Chamberlain and Haaga [19] highlight the fact that low unconditional self-acceptance is associated with depression and anxiety, as well as with low levels of self-esteem, happiness, and life satisfaction.

Knowing all these factors can help clinical psychologists contribute to the improvement of the quality of life of patients with RA. Starting from this premise, the pilot research was carried out in two stages. For the first stage, the objectives were (1) to identify the relationships that exist between the level of anxiety developed by patients, the frequency of automatic negative thoughts, and unconditional self-acceptance and (2) to capture the differences regarding these variables between people diagnosed with rheumatoid arthritis and people with no such medical history. In the second stage, based on the results obtained, a pilot strategy was devised to create a support and psychological counseling group for people diagnosed with rheumatoid arthritis, followed by a new evaluation of the studied variables after a period of six months.

## 2. Methods

### 2.1. Participants

The first stage of the study was carried out during year 2012 within the County Hospital located in Reşiţa, on the psychologist's initiative, approved by the chief of the hospital's psychology department. The sample of the research included 50 participants: one sample made up of 25 people diagnosed with rheumatoid arthritis (RA) for at least 5 years, frequently hospitalized and registered at the County Hospital (with an average age of 49.43), and a witness sample made up of 25 in-good-health people with no such medical history or other chronic diseases (with an average age of 51.71).

### 2.2. Procedure

The participation in the study was voluntary; the subjects were given instructions regarding the study, its purpose, and benefits, followed by their verbal agreement. They were asked to fill out three licensed questionnaires, part of a battery of questionnaires adapted to the Romanian population [20]. A semistructured interview was also necessary in order to gather information about disease history and patients' quality of life. People within the sample diagnosed with rheumatoid arthritis for at least 5 years (11 man and 14 women) were hospitalized for two weeks. Participants in the witness sample (10 man and 15 women) were selected randomly, with the main condition being the absence of this disease in their medical history.

### 2.3. Measures

The three questionnaires used in the first stage of this pilot research are part of a battery of questionnaires adapted to the Romanian population and distributed under license [20]. (1) The Hamilton Anxiety Scale, developed by Hamilton (2007) [21], is used to evaluate the severity of anxiety symptoms in children and adults. The scale includes 14 items for evaluating cognitive, emotional, and, mainly, somatic components (physical complaints related to anxiety), an evaluation based on the patients' current state of health and following a semistructured interview that includes questions regarding the presence of anxiety symptoms; the answers are given on a 5-step scale (0: no anxiety, 4: severe anxiety); (2) the Automatic Thoughts Questionnaire, developed by Hollon and Kendal (2006) [22], is made up of 15 items that measure the frequency of dysfunctional and irrational automatic negative thoughts about oneself; the answers are given on a 5-step Likert scale (1: never, 5: almost always), according to the frequency of these thoughts during the last four weeks; (3) the Unconditional Self-Acceptance Questionnaire, developed by Chamberlain and Haaga (2007) [23], includes 20 items that measure unconditional self-acceptance, regardless of behavior, achievement, approval, respect, or love from others; the answers are given on a 7-step Likert scale (1: almost always false, 7: almost always true), according to the subjects approval or disapproval of the given statements. Individual semistructured interviews were also carried out in order to gather information about the rheumatoid arthritis-related medical history and the participants' quality of life.

### 2.4. Analysis

The data obtained by applying the questionnaires was statistically processed using the SPSS version 16 program. In order to achieve the objectives set for the first stage of the research, correlations were calculated using the Bravais-Pearson coefficient; as data distribution was not normal, and the samples sizes were less than 30 participants, for establishing the differences, the nonparametric Mann-Whitney *U* test was used for comparing two independent samples [24].

## 3. Results 

For the first stage, the first objective of the pilot study was to (1) identify the relationships between the level of anxiety developed by patients, the frequency of automatic negative thoughts, and unconditional self-acceptance.  Table 1 shows the positive correlations obtained between psychological anxiety and automatic negative thoughts, highlighting the fact that an increase in psychological anxiety is linked to a higher frequency of automatic negative thoughts; the negative correlations obtained between psychological anxiety and somatic anxiety, respectively, and unconditional self-acceptance, indicating that higher psychological and somatic anxiety are tightly related to lower self-acceptance; and a negative relationship between automatic negative thoughts and unconditional self-acceptance, indicating that higher frequency of automatic negative thoughts is linked to lower unconditional self-acceptance.

The second objective aimed at highlighting the differences between the level of anxiety, the frequency of automatic negative thoughts, and unconditional self-acceptance in the case of people diagnosed with rheumatoid arthritis as compared to people not suffering from this disease, in order to develop counseling and support programs—in the second stage of the study.  Table 2 shows the existing differences between the two samples in terms of psychological and somatic anxiety—indicating a higher level of psychological and somatic anxiety in the case of subjects diagnosed with RA; a higher frequency of automatic negative thoughts in the case of subjects diagnosed with RA; and a higher level of unconditional self-acceptance in the case of subjects without RA.

## 4. Discussion 

The results obtained in this preliminary study are in conformity with the biopsychosocial model of the RA. Apart from anxiety and depression, associated with chronic suffering and intensified pain [11], relationships with two other types of psychological variables were also captured: automatic negative thoughts and unconditional self-acceptance.

According to the cognitive approach to depression, one's emotions and behaviors are determined by the way in which one interprets events, interpretation based on the circumstances in which the event occurs, on one's disposition, and on other past events that are part of one's personal experience [20]. The theory of automatic negative cognitions stresses the importance of structures that represent past experiences that influence the processing and organizing of current information [25], structures that were named automatic negative cognitions. These are cognitive products in the form of reasoning and prediction made by the individual about oneself, the world, and the future. Automatic negative cognitions are repetitive and escape immediate control [25]. They are activated quickly, allow the individual to save resources, and determine a quick categorization of new stimuli from the environment, favoring the update of complex scenarios [13]. The dysfunctional cognitive schemes can also be the source of cognitive distortions; they can favor a faulty or a preferential encoding of certain stimuli, omissions or errors, and inadequate behaviors [14]. Once activated, these negative automatic thoughts determine emotional and behavioral response to a stimulus or situation and allow biased information processing. Thus, dysfunctional patterns are reinforced, whereas adaptive but inconsistent patterns are suppressed [26]. The prevalence of automatic thoughts, that is, those dysfunctional cognitions that affect both the perception of oneself and external perception, has been associated with depression [26]. In a study carried out by Flett et al. [17], the authors also highlighted the existence of a link between low unconditional self-acceptance and symptoms of depression. At the same time, low unconditional self-acceptance is tightly correlated with low self-esteem and elevated levels of self-esteem liability and proneness to depression [27]. Self-esteem is part of those factors that are associated with mental health. A low self-esteem as a consequence of problems that arose in the past is associated with the presence of symptoms specific of depression [28]. Although there is a link between unconditional self-acceptance and global self-esteem, the two concepts do not overlap [27].

In the case of the study sample, one can notice the fact that an enhancement of psychological anxiety (mental nervousness and psychological distress) and somatic anxiety (physical complaints related to anxiety) is closely connected to a decrease in the patients' self-acceptance, and an increase in the frequency of automatic negative thoughts is followed by a decrease in unconditional self-acceptance. In terms of differences regarding these variables between people with RA and people without RA, results indicate the presence of psychological and somatic anxiety and a higher frequency of automatic negative thoughts in the case of patients diagnosed with RA and a higher level of unconditional self-acceptance for patients without RA.

Based on the analysis of these results a pilot strategy was developed in order to create a support and psychological counseling group for people taking part in this study. The interventions focused on the action on psychological and somatic anxiety, on automatic thoughts associated with depression, and on unconditional self-acceptance. The strategy followed a general plan as well as an individualized one. Thus, for (1) the intervention on automatic thoughts associated with depression, the starting point was using techniques that would allow their identification, followed by applying techniques for altering automatic thoughts; for (2) the intervention on anxiety, on a general level the focus was on learning how to relax through the Schultz autogenous training, followed by the development of individualized plans for reducing anxiety where this measure was needed to a greater extent. All these activities aimed at changing maladaptive thought and behavioral patterns, offering the patients the opportunity to acquire tools for adapting to the situation and the disease [29]. Moreover, studies show that therapeutic interventions must firstly focus on automatic negative thoughts because these are the closest to conscious awareness [16]. After identifying, evaluating, and altering these thoughts, one can notice a decrease in depression as these thoughts are associated not only with depression, but also with coping styles [26]. After carrying out an evaluation of the subsequent progress that followed these programs, a new testing and adaptation of the support and psychological counseling strategy are intended, depending on the results obtained.

This undertaking was chosen due to the increasing interest in the patients' perspective on their own illness. Along with the traditional clinical approach interested in the presence and severity of joint inflammation and the end-organ damage, the evaluation of a third domain, that of the quality of life, carried out by the patients themselves, becomes very important [30].

## 5. Conclusion and Implication for Further Research

Although the current study is in line with the results of other research studies, a series of limitations must be noted. Firstly, one must note the small number of participants that were included in the study. Despite this fact, a number of differences were noticeable between the two samples, regarding the variables investigated. Although many clinical research studies aim at identifying the presence of depression symptoms in such situations, the current study did not employ a questionnaire that would allow this. The three psychological variables investigated—anxiety, automatic negative thoughts, and unconditional self-acceptance—were, however, explained by referring to their relationship with depression-specific symptoms. However, the portfolio of questionnaires focused on other variables as well, such as decision making ability or the subjects' attitudes and general beliefs. Although differences between the two samples were obtained, these were not analyzed in this stage of the research, with the main concern being the creation of a support and psychological counseling group for people with RA. Despite these limitations, the study also bears potentially important clinical implications. The results highlight the presence to a greater extent of anxiety and automatic negative thoughts, along with reduced unconditional self-acceptance among people with RA. The intervention on these variables through support and counseling can lead to reducing anxiety and depression, to altering the coping styles, and, implicitly, to improving the patients' quality of life. Also, developing such programs that allow the patients' education, from providing information and learning common nursing techniques to using cognitive-behavioral strategies within these programs, can become an important component of active management programs for RA [31].

## Figures and Tables

**Table 1:** Correlation coefficients between psychological and somatic anxiety, automatic negative thoughts and unconditional self-acceptance.

Variables	1	2	3	4
(1) Psychological anxiety	1.00			
(2) Somatic anxiety	—	1.00		
(3) Automatic negative thoughts	.530**	—	1.00	
(4) Unconditional self-acceptance	−.404**	−.397**	−.594**	1.00

Note: *N* = 50; **correlation coefficient significant at *p* < .01.

**Table 2:** Differences between psychological and somatic anxiety, automatic negative thoughts, and unconditional self-acceptance.

Variables	Participants	Mean rank	Mann-Whitney *U *	*Z *	*p *
Psychological anxiety	Sample with RA	34.28	93.000	−4.277	.000
	Sample without RA	16.72			
Somatic anxiety	Sample with RA	35.14	71.500	−4.692	.000
	Sample without RA	15.86			
Automatic negative thoughts	Sample with RA	29.88	203.000	−2.128	.033
	Sample without RA	21.12			
Unconditional self-acceptance	Sample with RA	19.50	162.500	−2.913	.004
	Sample without RA	31.50			

Note: *N* = 50 (25 participants with RA and 25 participants without RA).
